# Partial nicotinic acetylcholine (α4β2) agonists as promising new medications for smoking cessation

**DOI:** 10.4103/0253-7613.44150

**Published:** 2008-10

**Authors:** J. Singh, Salil Budhiraja

**Affiliations:** Department of Pharmacology, Pt. B. D. Sharma PGIMS, Haryana, India

**Keywords:** Cytisine, partial agonist α4β2 nAChR, smoking cessation, SSR591813, varenicline

## Abstract

**Objective:**

To review the pharmacology, clinical efficacy and safety of partial agonists of α4β2 nicotinic acetylcholine receptor.

**Data Sources:**

Primary literature and review articles were obtained via a PUBMED search (1988-August 2006) using the key terms smoking cessation, partial agonist alpha4beta2 nicotinic acetylcholine receptor, varenicline, cytisine and SSR591813. Additional studies and abstracts were identified from the bibliographies of reviewed literature.

**Study Selection and Data Extraction:**

Studies and review articles related to varenicline, cytisine and the partial agonist alpha4beta2 nicotinic acetylcholine receptor were reviewed.

**Data Synthesis:**

Smoking is widely recognized as a serious health problem. Smoking cessation has major health benefits. According to the US Public Health Services, all patients attempting to quit smoking should be encouraged to use one or more effective pharmacotherapy. Currently, along with nicotine replacement therapy, bupropion, nortriptyline and clonidine, are the mainstay of pharmacotherapy. More than ¾ of patients receiving treatment for smoking cessation return to smoking within the first year. Nicotine, through stimulating α4β2 nAChR, releases dopamine in the reward pathway. Partial agonist of α4β2 nAChR elicits moderate and sustained release of dopamine, which is countered during the cessation attempts; it simultaneously blocks the effects of nicotine by binding with α4β2 receptors during smoking. Recently, varenicline, a partial agonist at α4β2 nAChR, has been approved by the FDA (Food and Drug Administration) for smoking cessation.

**Conclusion:**

Partial agonist α4β2 nAChR appears to be a promising target in smoking cessation. Varenicline of this group is approved for treatment of smoking cessation by the FDA in May 2006.

## Introduction

Smoking is widely recognized as a serious health problem, which is increasing in prevalence across the world. Smoking is a high risk factor for coronary heart disease, chronic obstructive pulmonary disease, cancers and various other health related problems.[1] According to a recent study, about five million premature deaths in the world are attributed to smoking. Worldwide, it is estimated that the prevalence of smoking averages 33% of the population aged 15 years and older. According to current estimates, there are 1.1 billion smokers worldwide.[1] The composition of tobacco smoke is complex, but it chiefly contains nicotine and tars and nicotine is the main active substance of tobacco products responsible for the positive reinforcement in humans and in animals.[2] Chronic tobacco use in humans or chronic administration of nicotine in rodents produces a state of “physical dependence” characterized by the occurrence of withdrawal syndrome.[2] Although, the prevalence of smoking is decreased, the lack of significant decline is alarming. This gives rise to an urgent need for increasing awareness on smoking cessation and for better pharmacotherapy for smoking cessation.

According to the US Public Health Services guidelines, all patients attempting to quit smoking should be encouraged to use pharmacotherapeutic agents, along with behavioral therapy. The use of pharmacotherapy in patients trying to quit smoking increases the likelihood of smoking cessation, as well as the chances of remaining free from smoking, after a long duration of abstinence. Currently, pharmacotherapies for smoking cessation are either nicotine replacement by patch, gum, lozenge, inhaler and nasal spray or bupropion, nortriptyline and clonidine.[3] Nicotine therapies are used as a substitution for tobacco smoking. It is believed that after long term use, it will reduce cigarette consumption by smokers.[4] Whereas, bupropion and nortriptyline are dopamine and norepinephrine uptake inhibitors and they increase the concentration of dopamine in the nucleus accumbens and may lead to the same positive reinforcing effect observed with nicotine.[5] Clonidine reduces the withdrawal effects of nicotine.[3] Combination therapies and long term medication for smoking cessation further improve the outcome.

The clinical use of nicotine as a therapeutic agent is severely limited by its cardiovascular and gastrointestinal side effects.[4] These therapies are effective, but the success rate is limited. Even the most successful smoking cure clinics, using a combination of psychological and pharmacological treatments, achieve a success rate of less than 30% after one year of abstinence.

### Data source

A literature review was conduced, consisting of a PUBMED database search of English language articles using the search terms smoking cessation, partial agonist alpha4beta2 nicotinic acetylcholine receptor, varenicline, cytisine and SSR591813. The bibliographies of these articles were then reviewed for inclusion of other relevant articles not included in the PUBMED search. Data from the press release of Pfizer provided additional information.

### Neurobiology of nicotine dependence

**Figure 1:**
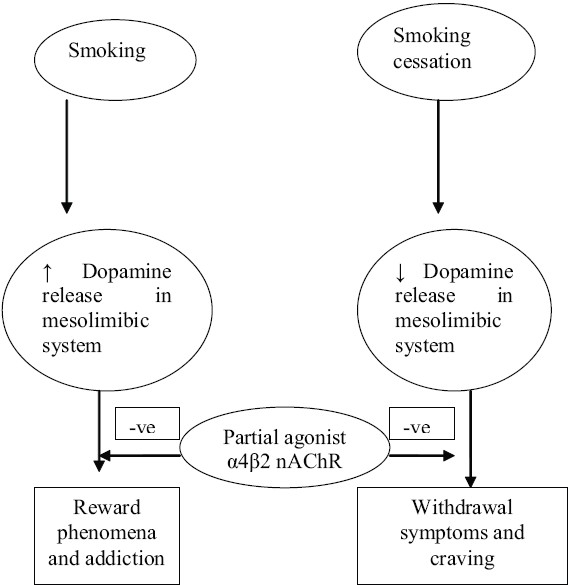
The role of partial agonist α4β2 nAChR receptor on nicotine addiction

Although the molecular mechanisms that contribute to nicotine dependence are poorly understood, the neuromodulatory role of nicotinic cholinergic system in the central nervous system is principally implicated for it.[6] Numerous other neural pathways and neurotransmitters, including GABA, serotonin, glutamate, noradrenaline, endogenous opioid, and corticotropin releasing hormone, have also been shown to play a role in nicotine dependence [Figure 1].[7]

Nicotine induces its central pharmacological effects by acting on nicotinic acetylcholine receptors (nAChR), which are ligand gated ion channels comprising five subunits. To date, molecular cloning techniques have identified 16 known receptor subunits (α_(1-10),_ β(1-4)_,_ γ (ε in adult) and δ). An adult skeletal muscle has the composition (α1)_2_ β1γ ε, while in neuronal nAChR α4β2, α3β4, α7 predominates.[8] The distribution of the various subunits in the rat brain has shown distinct expression patterns. The majority of the neuronal nAchRs fall into two categories: those that bind agonist with low affinity, presumably homomeric alpha7 receptors, and those bind agonist with high affinity - α4β2 nAChRs, which account for >90%.

Nashmi *et al.*[8] elaborated the CNS localization of neuronal nicotinic receptors. Several findings suggest that the α4β2 nAChR subtypes are present in nucleus accumbens and play a major role in the reinforcing effects of nicotine.[8,9]

Nicotine self administration is reduced in rats pretreated with the selective α4β2 nAChR antagonist DHβE.[10] The dopamine hypothesis of drug addiction postulates that increased mesolimbic dopaminergic transmission is a common mechanism of action for drugs of abuse, including nicotine, morphine, ethanol and cocaine.[11] Others have also suggested that smoking produces reduction in global activity, activation of prefrontal cortex and thalamus, and an increase in the dopamine concentration in nucleus accumbens.[12] Nicotinic self administration is reduced by pretreatment with dopamine antagonist and also by lesions of mesolimbic dopamergic neurons.[13] In rats, nicotine increases extracellular levels of dopamine in limbic areas, particularly in the shell of the nucleus accumbens and in the bed nucleus of stria terminalis, an area which is a part of the extended amygdale and which is interconnected with the nucleus accumbens and the ventral tegmental area.[14]

Nicotine abstinence reduces dopamine release and is associated with withdrawal symptoms and craving for nicotine.[6] The effects of nicotine on dopaminergic function could be mediated by the α4β2 nAChR located on neurons in the nucleus accumbens.[15] Mameli-Engvall *et al.*[16] suggested a hierarchical control of dopaminergic neuron firing patterns by nAChRs. They demonstrated the important role of β2 subunit of α4β2 for firing of the dopaminergic neurons and the release of dopamine from these neurons. Activation of β2-nAChR switches cells from a resting to an excited state, which is further tuned by other subunits, resulting in dopamine release. Several other studies have also suggested the importance of β_2_ subunits in reinforcing the effects of nicotine. Picciotto *et al.*[17] suggested that the α_4_β_2_ nAChR subtype has a major role in the reinforcing effects of nicotine and the β_2_ subunit is crucial in mediating the dopamine-releasing effects of nicotine, as indicated by the absence of striatal dopamine release in β_2_ subunit knockout mice treated with nicotine.

It has also been suggested that the transition from voluntary drug seeking to a compulsive habit might be brought about by long term potentiation or synaptic plasticity in striatum.[18] Several neural pathways/neurotransmitters other than dopamine (glutamate, GABA, endogenous opioids and endocannabinoid) are also implicated for tobacco dependence, but the exact role is not defined and this area still needs to be explored.[7]

### Effects of partial agonist at nAChR subtype α4β2 on nicotine dependence

Partial agonists of the alpha4beta2 nicotinic acetylcholine receptor are a new class of agents that are recently being studied for various neurological disorders such as Alzheimer disease, dementia[19] and attention deficit or hyperactivity disorder in adults.[20] Recently, the potential of the partial agonist at the neuronal acetylcholine receptor subtype α4β2 for smoking cessation has received considerable attention. Treatment for smoking cessation with a combination of nicotine replacement therapy and the nicotine antagonist mecamylamine was found to be more successful than replacement therapy alone, suggesting the role of partial agonist.[21]

Nicotine abstinence is associated with low levels of dopamine in the mesolimbic area of the brain. It is also associated with withdrawal symptoms and craving for nicotine. Partial agonists of the α4β2 nAChR, through intrinsic activation, produce a moderate and sustained increase in dopamine levels in the reward pathway. They counteract the low dopamine levels encountered during cessation attempts, which are responsible for a relapse. Additionally, by binding to the α4β2 nAChR, the partial agonist will prevent binding of nicotine and protect the smokers from the reward effect produced by dopaminergic activation while smoking.[22] [Figure 1] Foulds [23] elaborated the basis of partial agonist α4β2 nAChR in the treatment of nicotine dependence.

Cytisine is a plant alkaloid found in various parts of numerous plants, especially those belonging to the leguminosae family.[24] It has partial agonistic activity at α4β2 nAChR, with low efficacy.[22] When given alone subcutaneously, at a dose of 5.6 mg/kg, the drug increases the dopamine turnover with an efficacy amounting to 40% of the nicotine central effect.[22] Synthetic compounds are derived from carbon analogue of cytosine, but these also have low potency and low efficacy at α4β2 nAChR.[25]

Compounds under development include TC-1734, ABT-089, TC-2559, and SSR591813. They have high efficacy as a partial agonistic activity.[19,20,22,25,26] Recently, varenicline has been approved by the FDA for the treatment of smoking cessation.[27] Ongoing studies may provide further information regarding safety and long term abstinence from smoking by varenicline. This review focuses on the effects of partial agonist at nAChR subtype α4β2, for the cessation of smoking. We will first summarize the animal models used to assess subjective and rewarding/reinforcing effects of smoking and then discuss the preclinical and clinical findings related to the smoking cessation effect of partial agonist at nAChR subtype α4β2.

### Animal model for studying effects of nicotine

A variety of animal models are available for studying nicotine dependence. A systematic evaluation of these procedures by Stolerman[28] revealed that animal studies of the behavior pharmacology of nicotine dependence show good interspecies consistency.

Nicotine dependence has been evaluated using animal models for the subjective effects of nicotine (drug discrimination), its rewarding/reinforcing properties [intravenous drug self administration, conditioned place preference (CPP)], the influences of environment factors on nicotine seeking behavior (reinstatement of extinguished drug seeking behavior) and the withdrawal states associated with the abrupt termination of nicotine action (administration of mecamylamine after chronic exposure).

The animal's ability to perceive and identify the characteristic effects of nicotine is thought to play a role in nicotine seeking, encouraging the development of this behavior and directing it towards nicotine rather than another.[29] An animal model for studying the subjective effects of nicotine (drug discrimination) has been developed. Animals are trained under a discrete trial schedule of food pellet delivery, to respond on lever pressing after an injection of nicotine, and another lever after an injection of vehicle. Once the animals learn to reliably make this discrimination, the subjective effects of nicotine can be compared.[29]

Nicotine is a positive reinforcer in animals. Positive reinforcement, pressing lever, results in the delivery of the drug injection, serving as an important model to study the reinforcing effects of nicotine. Animals learn to emit a specific response (pressing of lever) in order to receive the intravenous injection of drug.[30] Either progressive ratio schedule (increase number of lever press after each dose) or fixed ratio schedule (fixed number of lever pressing necessary to obtain the i.v. dose) can be used.

Environmental cues are also important for nicotine self administration behavior. Such stimuli, through Pavlovian conditioning, can induce and maintain drug seeking behavior.

CPP is another experimental animal model developed for assessing the rewarding effects of nicotine. In animals, nicotine has been shown to induce a preference for the box compartment repeatedly associated with the drug administration.[31]

Withdrawal state of nicotine can also be studied in the rodent model. Withdrawal is produced by the abrupt termination of drug action (administration of selective antagonists after chronic exposure).[32] The difficulty in developing such models using nicotine has been a limiting factor in understanding nicotine addiction.

### Preclinical trials

A number of preclinical studies have been conducted for studying the effect of partial agonists of α4β2 nAChR in *in vitro* and in rodent models. These studies have demonstrated that partial agonists of nAChR treatment were associated with reduction in nicotine binding to the α4β2 nAChR receptor while smoking, and moderate but sustained release of dopamine in mesolimbic pathways during cessation attempts.

Stuhmer[33] demonstrated the partial agonistic properties of varenicline in Xenopus oocytes, expressing the α4β2 nAChR. In the presence of nicotine (10 *µ*M), varenicline antagonized nicotine's effect in Xenopus oocytes model. Similarly, partial agonistic activity of varenicline was demonstrated by others. Varenicline binds with subnanomolar affinity to α4β2 nAChR and in *in vitro* functional patch clamp studies in HEK cells expressing nAChRs, which show that varenicline is a partial agonist with 45% of nicotine's maximal efficacy at α4β2 nAChR. In neurochemical models, varenicline has significantly lower (40-60%) efficacy than nicotine in stimulating[3] H-dopamine release from rat brain slices *in vitro*.[34] Similar results were also shown for cytisine. Papke *et al.*[35] demonstrated the partial agonistic activity of cytisine at α4β2 nAChR, with low efficacy. In their experiments, the responses of α4β2 injected oocytes to the application of 1 mM cytisine amounted only to 15% of the response to 1mM Ach. The coadministration of cytisine and Ach resulted in the reduction of the response to Ach.

Coe *et al.*[22] studied the effects of partial agonists, varenicline and cytisine on mesolimbic dopamine turnover in Sprauge-Dawley rats. They demonstrated that nicotine increased the dopamine turnover in mesolimbic area, whereas varenicline and cytisine have 30-40% of nicotine response for dopamine turnover. They further demonstrated the ability of cytisine and varenicline to attenuate nicotine's effect on the mesolimbic dopamine, when administered concurrently with nicotine. In comparative study, varenicline was more potent for blocking the central effects of nicotine. Wang *et al.*[26] studied the role of TC-2559, a partial agonist of α4β2 nAChR, on dopaminergic neurons of VTA. They reported that TC-2559 increased both the firing and bursting activities of VTA dopaminergic neurons, similar to nicotine. They also demonstrated that excitation evoked by TC-2559 can be reversed by selective antagonist of α4β2 nAChR (DHβE). In a brain microdialysis study, SSR591813 produced an increase in dopamine levels, which is approximately two-fold less than nicotine, and pretreatment with SSR591813 completely blocked the effect of nicotine.[36]

In drug discrimination animal model, pretreatment with SSR591813 (10 mg/kg) antagonized the discriminative effects of the nicotine.[36] SSR591813 also prevented the withdrawal signs precipitated by mecamylamine, in nicotine dependent rats, and partially blocked the discriminative cue of acute precipitated withdrawal.[36] Cohen *et al.*[36] demonstrated that in nicotine self administration model, SSR591813 (20 mg/kg, i.p.) significantly reduced the number of nicotine infusion and lever pressing.

In conclusion, *in vitro* and *in vivo* studies suggest that partial agonist of α4β2 nAChR would moderately increase the dopamine level in the mesolimbic system, attenuating the withdrawal symptoms, and, on the other hand, it should minimize the addictive effects of nicotine by decreasing the dopamine level. The lower efficacy of partial agonist of α4β2 nAChR in causing dopamine release, as compared with nicotine, suggests that these would be significantly less addictive.

### Clinical trials

Varenicline has undergone full scale clinical development and has been recently approved by the US FDA for the treatment of nicotine addiction. The efficacy of the drug in smoking cessation was demonstrated in seven clinical studies, including three comparative trials with bupropion. Wu *et al.*[37] systematically reviewed and meta analysed the effectiveness of different therapies (nicotine replacement therapy, bupropion and varenicline) for smoking cessation. He evaluated four studies to assess the effect of varenicline and found that varenicline was superior to nicotine replacement therapy. In two multicenter, randomized, placebo controlled trials, more than 2500 smokers were recruited and treated either with placebo or varenicline (0.5 to 1.0 mg once or twice daily) for 12 weeks and follow up was done up to one year. Efficacy measured by carbon monoxide (CO), confirmed four week continuous quit rates and continuous abstinence as self report. These trials showed that varenicline is efficacious for smoking cessation.[38,39]

In two randomized, double blind, multicenter trials involving more than 2000 smokers, varenicline, bupropion sustained release and placebo were given for 12 weeks and then followed up to 52 weeks. In the studies, the number of smokers who were treated with varenicline and thereafter quit smoking was significantly more than those treated with bupropion and placebo. Carbon monoxide confirmed continuous abstinence rate during the nine to 12 weeks of the treatment was 44% for varenicline, which was better than for sustained release bupropion (30%) or placebo (18%). The continuous abstinence rate from week 9 through week 52 was 22-23%, as against 15-16% for bupropion and 8-10% for placebo.[40,41]

In other randomized double blind studies with placebo and active treatment controlled, healthy smokers, 1023 subjects were screened and 638 were randomized to get varenicline (0.3, 1,2 mg/day), bupropion 300 mg/day and placebo for seven weeks, with follow up for one year. The studies showed that the continuous quit rates were significantly higher for varenicline treated smokers than for placebo (48% vs 17.1%). The varenicline treated group was also more prone to quitting smoking during the duration of follow up (14.4% vs 4.9%).[42] The adverse effects were relatively common, but they did not result in significantly higher discontinuation rate than with placebo.[40,41] Two randomized, placebo controlled trials were conducted on Asian population (Japanese, Taiwan and Korea), to determine the efficacy and tolerability of varenicline. A double blind and parallel group study was conducted on Japanese population with 0.25, 0.5, 1.0 mg BID dose and treatment was given for 12 weeks; follow up was done up to 52 weeks. In a study on Taiwan and Korean population, varenicline 1 mg BID was given for 12 weeks and follow up was done for 12 weeks. Both the studies demonstrated that varenicline was efficacious and was well tolerated in Asian population.[43,44]

One multicenter, double blind, randomized trial was done to assess the long term safety of varenicline. Two hundred and fifty one subjects were randomized to receive varenicline and 126 to receive placebo for 52 weeks. The adverse events were recorded. The study demonstrated that varenicline 1mg BID was safe and that it could be safely administered for up to one year.[45]

Cytisine, a partial agonist of α4β2 nAChR, is widely used in eastern and central Europe for smoking cessation. Many clinical studies on cytisine as a treatment in smoking cessation have suggested that the drug is efficacious and safe; however, these studies do not conform to modern standards and should be interpreted with caution.[46]

Taken together, the reports of clinical studies suggest that partial agonists of α4β2 nAChR represent promising alternatives to the agents being currently used for the therapy of smoking cessation.

### Pharmacokinetics

The maximum plasma concentrations of varenicline (C_max_) after 1.0 mg oral dose of varenicline is 3.2 to 4.7 ng/ml and achieved within two to seven hours. The elimination half life of varenicline is approximately 17±3 h after 1.0 mg oral dose. Varenicline undergoes minimal metabolism, with 92% excreted unchanged in urine. Metabolites occur via N-carbamoyl glucuronidation and oxidation.[47] Renal elimination of varenicline is primarily through glomerular filtration, along with active tubular secretion. Oral bioavailability of varenicline is unaffected by food or time of day dosing. *In vitro* studies demonstrated that varenicline does not inhibit or induce cytochrome P450 enzyme.[48]

### Adverse effects

In Phase 2 and 3 studies, the treatment discontinuation rate due to adverse events with varenicline was 12%, as compared to 10% for placebo. The most common adverse events reported in the premarketing development of varenicline were nausea, sleep disturbance, constipation, flatulence, and vomiting. There have been reports of neuropsychiatric syndromes (depressed mood, agitation, changes in behavior, suicidal ideation and suicide) in post marketing surveillance in patients attempting to quit smoking while taking varenicline.[48]

### Indications and contraindications

The drug is indicated as an aid to smoking cessation treatment and is contraindicated in persons hypersensitivite to it.

### Drug interactions

No clinically significant pharmacokinetic drug-drug interactions have been identified. Cimetidine increases the systemic exposure of varenicline by 29%.[48]

Current status of partial agonist of α4β2 nicotinic receptor for smoking cessation

Despite advances in the understanding of the neurobiological and the behavioral mechanisms that lead to nicotine dependence, effective treatment is still lacking. Besides, the treatment that is currently available is unsatisfactory; the relapse rate is very high. Most of the patients relapse even after the best type of treatment.

Alpha4beta2 nicotinic acetylcholine receptor represents a potential target for smoking cessation. By reducing the motivational effects of nicotine and preventing the withdrawal symptoms during smoking cessation, partial agonist of α4β2 nicotinic acetylcholine might provide an effective means for preventing relapse to smokers. Currently varenicline, partial agonist of the α4β2 nicotinic acetylcholine, has been approved by the FDA on 11 May, 2006 for smoking cessation.
